# Brucine Entrapped Titanium Oxide Nanoparticle for Anticancer Treatment: An *In Vitro* Study

**DOI:** 10.1155/2024/4646855

**Published:** 2024-03-18

**Authors:** Rashed M. Almuqbil

**Affiliations:** Department of Pharmaceutical Sciences, College of Clinical Pharmacy, King Faisal University, Al-Ahsa 31982, Saudi Arabia

## Abstract

*Backgroundand Objective*. The public's health has been seriously threatened by cervical cancer during recent times. In terms of newly diagnosed cases worldwide, it ranks as the ninth most prevalent malignancy. Multiple investigations have proven that nanoparticles can effectively combat cancer due to their small dimensions and extensive surface area. In the meantime, bioactive compounds which are biocompatible are being loaded onto nanoparticles to promote cancer therapy. The current study investigates the anticancerous potential of Brucine-entrapped titanium oxide nanoparticles (TiO_2_ NPs) in cervical cancer cell line (HeLa). *Materials and Methods*. The physiochemical, structural, and morphological aspects of Brucine-entrapped TiO_2_ NPs were evaluated by UV-visible spectrophotometer, Fourier transform-infrared spectroscopy (FT-IR), dynamic light scattering (DLS), scanning electron microscopy (SEM), and energy dispersive X-ray (EDAX). The cytotoxic effect against the HeLa cell line was assessed using a tetrazolium-based colorimetric assay (MTT), a trypan blue exclusion (TBE) assay, phase contrast microscopic analysis, and a fluorescence assay including ROS and DAPI staining. Furthermore, estimation of antioxidant markers includes catalase (CAT), glutathione (GSH), and superoxide dismutase (SOD). *Results*. The UV spectrum at 266 nm revealed the formation of TiO NPs. The FT-IR peaks confirmed the effective entrapment of brucine with TiO_2_ NPs. The average size (100.0 nm) of Brucine-entrapped TiO_2_ NPs was revealed in DLS analysis. The micrograph of the SEM revealed the formation of ellipsoidal to tetragonal-shaped NPs. The Ti, O, and C signals were observed in EDAX. In MTT assay, Brucine-entrapped TiO_2_ NPs showed inhibition of cell proliferation in a dose-wise manner and IC_50_ was noticed at the concentration of 30 *µ*g/mL. The percentage of viable cells gradually reduced in the trypan blue exclusion assay. The phase contrast microscopic analysis of Brucine-entrapped TiO_2_ NP-treated cells showed cell shrinkage, cell wall deterioration, and cell blebbing. The intracellular ROS level was increased in a dose-wise manner when compared to control cells in ROS staining. The condensed nuclei and apoptotic cells were increased in treated cells, as noted in DAPI staining. In treated cells, the antioxidant markers such as CAT, GSH, and SOD levels were substantially lower compared to the control cells. *Conclusion*. The synthesized Brucine entrapped TiO_2_ NPs exhibited remarkable anticancer activity against the HeLa cell line.

## 1. Introduction

Chemotherapeutic anticancer treatment involves the use of synthetic chemicals that inhibit cancer cell proliferation or tumor growth, which can be combined or taken alone. The primary negative implications of chemotherapy are its inability to target cancer cells and harm healthy cells [1, 2]. In order to prevent adverse effects, plant compounds such as Vinca alkaloids, taxanes, epipodophyllotoxins, camptothecins, genistein, and quercetin or rutin have been used in cancer treatment [3]. Though it was successful in inhibiting cancer cells, the majority of compounds showed restrictions in their solubility, bioavailability, penetration, and stability which made it difficult to translate into clinical trials. In this way, the advantages of both nanotechnology and natural products could be combined by delivering natural products through nanoparticle drug delivery systems [4].

TiO_2_ NPs are widely used as a white pigment in cosmetic products like sunscreen, powder, and eyeshadow due to their high availability and low toxicity. When compared to other nanoparticles (such as copper oxide, zinc oxide, and manganese oxide), TiO_2_ nanomaterials are less hazardous and biocompatible [5, 6]. Comparing TiO_2_ nanoparticles to other unstable (degradable) nanomaterials in an aqueous solution, the latter's long-term stability in biological settings is another benefit that can prevent the loaded biomolecules from denaturing. TiO_2_ NPs due to their high refractive index and UV absorbing capability, are used in sunscreen lotion, paint, food coloring, and personal care products [7–11]. The formation of hydrogen peroxide (H_2_O_2_) and hydroxyl free radicals by titanium dioxide (TiO_2_) nanoparticles has been demonstrated to have anticancer and anti-inflammatory effects on mammalian cells, releasing reactive oxygen species (ROS) [12, 13]. A key bioactive alkaloid produced from *Strychnos nux-vomica* is called brucine (2,3-dimethoxy strychnidin-10-one). The anticancer and anti-inflammatory properties of brucine can also treat central nervous system toxicity [14]. In addition, a recent study has suppressed cervical cancer cells (ME-180) through apoptosis through anti-inflammatory and antiproliferative activity via the PI3K/AKT/mTOR signalling pathway [15], while the induced apoptotic effect by HepG2 through Bcl-2 and Ca^**2+**^ involved mitochondrial pathway of brucine was reported earlier [16]. The primary objective of the present investigation was to employ a nanodelivery platform for evaluating the anticancer activity of brucine loaded on titanium oxide nanoparticles in cervical cancer cells (HeLa).

## 2. Materials and Methods

### 2.1. Preparation of TiO_2_+ Brucine

Titanium dioxide was reduced to TiO_2_ nanoparticles and coated with brucine as per the protocol described earlier [17] with the required modifications. Titanium dioxide (0.1 M) was weighed for 250 ml and stirred continuously. 5 molars NaOH were added to the content until it reached pH 12, and then stirring was continued for overnight. 0.25% of brucine was added to the solution and stirred for 3 hours for the synthesis of brucine-entrapped TiO_2_ NPs. Then the mixture was kept for sonication followed by centrifugation at 5000 rpm for 10–15 minutes. The pellet was dried in a hot air oven at 50–60°C for 1-2 hours. The dried powder was stored for further analysis.

### 2.2. Characterization of TiO_2_+ Brucine

The basic technique of characterization to analyze the nanoparticle reduction confirmation at the primary level through surface plasmon resonance (SPR) which is recorded by a UV-visible spectrometer in the wavelength range of 200 to 800 nm by UV-3,600 Shimadzu, Japan. Fourier transform IR (FT-IR) spectra were employed to investigate the active functional groups in the TiO_2_ nanoparticle and the degree of conjugation of the bioactive compound brucine with the TiO_2_ nanoparticle using Perkin Elmer spectrophotometer (Waltham, United States of America) in the wavelength range of 400 cm^−1^ to 4000 cm^−1^. The dynamic light scattering was carried out by utilizing Zetasizer (nanoparticle SZ-100, Horiba, Kyoto, Japan) [18]. The surface morphological analysis was carried out by electron microscopy [19] and elemental composition was analyzed by energy dispersive X-ray (Zeiss MultiSEM505 tandem EDAX equipment, Jena, Germany).

### 2.3. Cell Culture

The cervical cancer cell line (HeLa) was procured from ATCC, USA. The cells were grown using DMEM media with 10% fetal bovine serum and with 2% antibiotic solution. The cells were grown in an optimized condition and subcultured for further analysis.

#### 2.3.1. Cytotoxicity and Trypan Blue Assay

The primary screening was performed to investigate the anti-cancer potency of brucine entrapped TiO_2_ NPs on the cervical cancer cell line (HeLa) through the MTT (cytotoxicity) assay and the Trypan Blue Exclusion (TBE) assay (viability). For the cytotoxicity assay, the cells were seeded in 96 well cell culture plates at a preferable count (10000 cells/well) [20]. At 70–80% confluency, the various concentrations of brucine entrapped TiO_2_ NPs were added to each well from 0.5 mg/ml to 0.002 mg/ml by the serial dilution method, and the untreated was kept as a control. The plate was incubated for 24 hours, and then MTT dye was added to each well followed by 4 hours incubation. The complex formed was dissolved using DMSO and read at 490 and 630 nm. The cytotoxicity was expressed as % inhibition, which was calculated by percentage of inhibition = 1−[[*A*490–*A*630]_sample_/[*A*490–*A*630]_control_] × 100.

The cell viability analysis was performed using the TBE assay. For this study, the cells were seeded in 6 well plates based on the requirement of 1 × 10^4^ cells/well and incubated for 24–48 hours. Then, the cells were treated with brucine entrapped TiO_2_ NPs with different dosage ranges. The cells were collected by trypsinization after 24 hours of incubation. Trypan blue dye 40 *µ*l was added to 10 *µ*l of cells. The live and dead cells were counted using a hemocytometer, and the count was recorded [21]. The viability percentage was calculated by, % viability = No. of viable cells/total no. of cells × 100.

#### 2.3.2. Cytomorphological Changes Assessments

The synthesized brucine-entrapped TiO_2_ NPs of various concentrations 15, 30, and 60 *µ*g/mL were added to the HeLa cell line to investigate the gross morphometric changes. The treated and untreated cells were incubated for 24 hours, and the cytomorphological changes were examined under a phase contrast microscope, and the images were photographed (Olympus, Tokyo, Japan) [22].

### 2.4. Fluorescence Assay

#### 2.4.1. ROS Staining

The primary marker to study the status of apoptosis is reactive oxygen species level. To investigate the level of ROS, the DCFHDA method was employed, as described earlier [23]. Briefly, HeLa cells were seeded in a 24-well culture plate and treated with various dosages of brucine entrapped TiO_2_ NPs for 24 hours. After treatment, the untreated and treated cells were washed with PBS and incubated with DCFHDA dye (10 *µ*M) for 30 minutes at 37°C. Then, the dye was removed by washing, and the images were taken under the fluorescence microscope.

#### 2.4.2. DAPI Staining

The apoptotic potency of brucine-entrapped TiO_2_ NPs towards the HeLa cell line was studied using the DAPI staining method. HeLa cells were seeded in a 24-well cell culture plate. The cells were treated with brucine-entrapped TiO_2_ NPs of different concentration 15, 30, and 60 *µ*g/mL, and then the control and treated groups were washed with chilled PBS buffer and fixed using chilled methanol for 10 minutes. Then, using 4% formaldehyde, the cells were permeabilized and stained with DAPI dye. Followed by staining the cells were washed and the images were photographed using a fluorescence microscope [24].

### 2.5. Antioxidant Markers

#### 2.5.1. Estimation of the Catalase Assay

Brucine-entrapped TiO_2_ NP-treated and untreated cells were subjected to catalase estimation. The catalase protocol was employed with reference to the reported method with slight changes [25]. To the 0.5 ml of treated and untreated cell suspension, 1 ml of phosphate buffer (0.01 mM, pH 7.0), 200 *µ*l of 30% hydrogen peroxide, and 400 *µ*l of water were added and incubated at room temperature for 10 minutes in the dark. Ammonium molybdate (32.4 mM) 1 ml was used to stop the enzymatic reaction, and the complex was read at 405 nm spectrometrically. The catalase activity was expressed as KU/L.

#### 2.5.2. Determination of Reduced Glutathione Analysis (GSH)

The estimation of reduced glutathione was performed based on the protocol suggested earlier with minor modifications [26]. The treated and control cells were collected by trypsinization and centrifuged. The obtained cell pellet was homogenized using 0.1 M potassium phosphate buffer (pH 7.4). 0.5 ml of cell homogenate was added to 0.5 ml of sulfosalicylic acid (4%) and kept in incubation for 1 hour at 4°C. Then, the content was centrifuged and from the supernatant, 0.033 ml was taken and added with 0.9 ml of potassium phosphate buffer (0.1 M, pH 7.4) and 0.066 ml of DTNB (di thiobis 2-nitrobenzoic acid). The coloured complex formed was read at 412 nm, and the reduced glutathione was expressed as *µ*M sulfhydryl units.

#### 2.5.3. Superoxide Dismutase Radical Scavenging Activity (SOD)

The predominant parameter to investigate antioxidant status is to estimate the percentage release of superoxide dismutase. To estimate SOD, a simple method described in the literature with minor changes was adapted [27]. The brucine-entrapped TiO_2_ NPs were treated at different dosage ranges such as 15, 30, and 60 *µ*g/mL. The treated and untreated cells were subjected to SOD determination. The 200 *µ*l of cell suspension from the respective groups were mixed with 200 *µ*l NBT (0.08 mM), 400 *µ*l of NADH (0.25 mM), and 200 *µ*l PMS and kept for 10 minutes incubation in the dark and then the absorbance was taken at 560 nm using a UV spectrophotometer. The SOD release was calculated as, % SOD release = *A*0 − *A*1/*A*0 × 100, whereas *A*1—sample OD and *A*0—Control OD.

### 2.6. Statistical Analysis

The statistical analysis was performed using GraphPad Prism version 8.1. All the experiments were performed as duplicate, and the values from the experiments were subjected to a one-way ANOVA, and the comparison between treated and control group was analyzed using Tukey's multiple comparison test. All the values expressed here is the mean and standard deviation. *P* < 0.001 was considered as significant for the study.

## 3. Results

### 3.1. Characterization of Brucine-Entrapped TiO_2_ NPs

**Figure 1:**
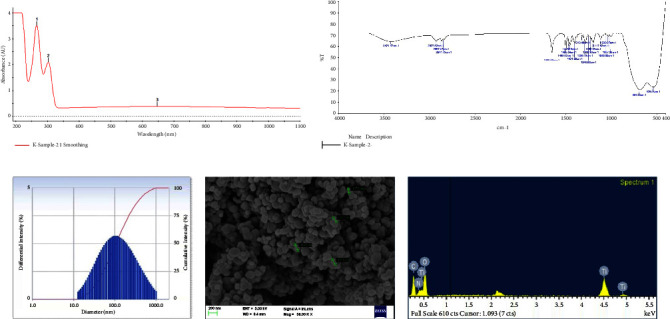
Characterization study of Brucine entrapped TiO_2_ Nanoparticle (a) Uv-Spectrum, (b) FTIR, (c) DLS, (d) SEM and (e) EDAX.

The optical characteristics of synthesized brucine-entrapped TiO_2_ NPs were analyzed using the UV–visible spectroscopy. The UV- Vis spectra at 266, 302, and 647 nm were observed (Figure 1(a)). The functional groups and chemical elements in the brucine entrapped TiO_2_ NPs were determined using the FT-IR spectrum (Figure 1(b)). The absorption band at 3421 cm^−1^ denotes the presence of alcoholic groups. The peaks at 2927 cm^−1^ and 2841 cm^−1^ are attributed to alkane group C-H stretching vibrations. The absorption band at 1653 cm^−1^, owing to the presence of brucine's carbonyl stretching vibrations. 1030 cm^−1^ indicates the presence of C-N stretching vibrations of amine. The absorption band between 400 cm^−1^ to 900 cm^−1^ is ascribed to the stretching vibrations of Ti-O and Ti-O-Ti. From DLS data, the average size of brucine-entrapped TiO_2_ NPs was found to be 100.0 nm (Figure 1(c)). In SEM analysis, ellipsoidal shapes with unevenly distributed tetragonal nanoparticles were observed (Figure 1(d)). From EDAX data, the elemental signals of Ti with a weight percentage of 38.94 and an atomic percentage of 16.22 were observed. Along with Ti signal, O signal with 42.57 weight percentage and 53.08 atomic percentage, C signal with weight percentage of 18.49 and atomic percentage of 30.70 was observed (Figure 1(e)).

### 3.2. MTT and TBE Assays

**Figure 2:**
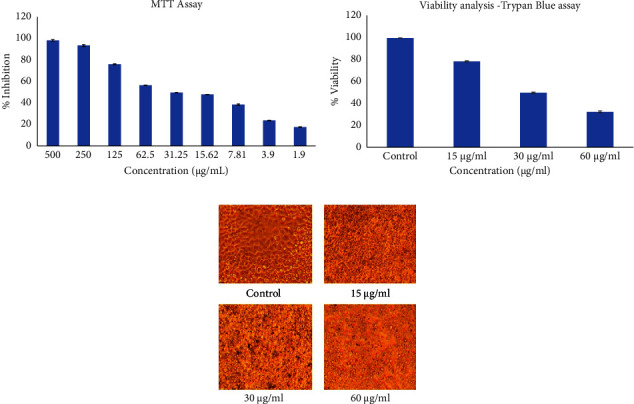
(a) Demonstration of the cytotoxic effects of Brucine entrapped TiO_2_ NPs in a dose-dependent manner (2–500 *µ*g/ml). The IC_50_ of Brucine entrapped TiO_2_ NPs on Hela cell lines was noticed at 30 *µ*g/ml concentration. (b) Representation of the percentage of cell viability. Trypan blue exclusion assay was used to examine the effect of Brucine entrapped TiO_2_ NPs on Hela cell lines. (c) Representation of the inhibitory action of Brucine entrapped TiO_2_ NPs was assessed by morphological alterations in Hela cell lines. The treated cells showed cell shrinkage, rupturing of cells, and cell rounding when compared to untreated cells. The distinct morphology was observed in control cells.

A cytotoxic investigation of brucine-entrapped TiO_2_ NPs was performed using 3-(4,5-dimethylthiazol-2-yl)-2,5-diphenyltetrazolium bromide dye which formed a yellow coloured formazan complex. The assay was performed in the range of 2–500 *µ*g/mL, and the IC_50_ value was obtained in 31.25 *µ*g/mL as 50.21% of inhibition. From the graph (Figure 2(a)), it is confirmed that the brucine-entrapped TiO_2_ NPs showed remarkable cytotoxic potency against cervical cancer cell line in a dose-dependent manner. At the low dosage of 1.9 *µ*g/mL itself, it showed 17.8% inhibition.

Similarly, the viability percentage was analyzed in the TBE assay using Trypan blue dye which binds with the cell wall of viable cells. The viability percentage was calculated in treated (15, 30, and 60 *µ*g/mL of brucine entrapped TiO_2_ NPs) and control groups showed 100, 78.24, 49.70, and 7.47 percentage for control, 15, 30, and 60 *µ*g/mL of brucine-entrapped TiO_2_ NPs, respectively (Figure 2(b)). The TBE assay results further confirm the cytotoxic potency of brucine-entrapped TiO_2_ NPs against the HeLa cell line.

### 3.3. Morphometric Analysis

Morphological changes of brucine-entrapped TiO_2_ NPs-treated and untreated HeLa cells were examined under phase contrast microscope (Figure 2(c)). The cells in the treated groups showed a gradual increase in the apoptotic percentage in a dose-dependent manner, as visualized by cell shrinkage, cell wall deterioration, and cell blebbing. The significant increase in the loss of cell adherence was also noticed as an increase in dosage range whereas control cells showed fined morphology with 100% confluency.

### 3.4. Fluorescence Assays

**Figure 3:**
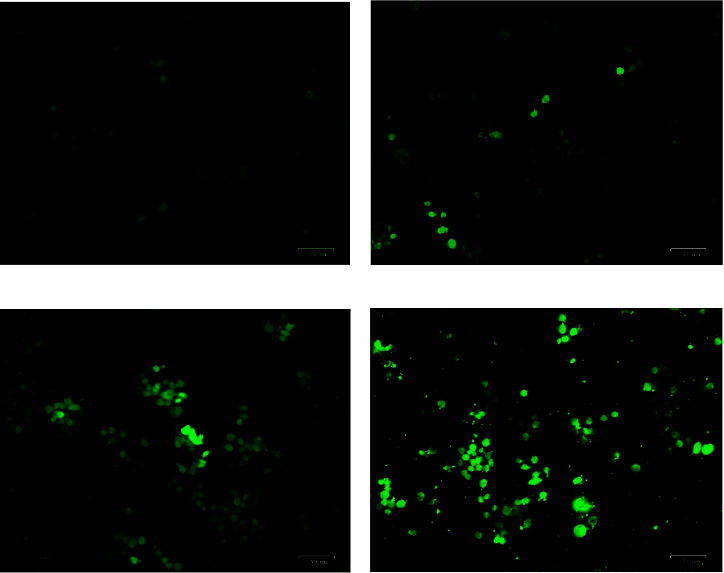
Representation of ROS staining by DCFHDA method. (a–d) represents Control, 15 *µ*g/ml, 30 *µ*g/ml and 60 *µ*g/ml respectively. ROS release was high in treated groups as dose dependent manner, whereas control showed negligible amount of ROS released.

Brucine-entrapped TiO_2_ NPs exposed cells were investigated by ROS staining and DAPI staining to confirm the root cause of antiproliferation effect. To understand the intracellular ROS release and apoptosis status of treated cells, these assays were employed.  Figure 3 shows the fluorescence images captured in the ROS assay stained by DCFHDA fluorophore. From the figure, it was clear that the intracellular ROS level was increased in a dose-dependent manner indicated by the increase in green fluorescence whereas control showed a very limited or negligible amount of intracellular ROS. The results indicate that brucine-entrapped TiO_2_ NPs create stressful circumstances when treated.

**Figure 4:**
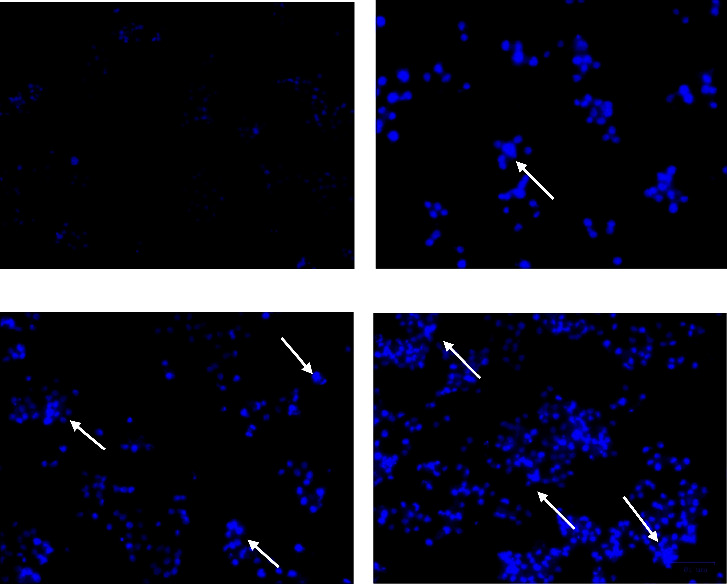
Representation of DAPI staining. (a–d) represents Control, 15 *µ*g/ml, 30 *µ*g/ml and 60 *µ*g/ml respectively. DAPI staining colour intensity was high in treated groups as dose dependent manner indicates the increasing count of damaged cells, whereas control showed very less staining indicates healthy cells. (White arrow indicates condensed nuclei, cell blubbing and ruptured cells).

In DAPI staining, the image (Figure 4) showed treated and untreated photographs of HeLa cells stained with DAPI dye, which readily binds with the condensed nuclei. The gradual increase of blue fluorescence in a dose-dependent manner strongly confirms the cytotoxic property of brucine-entrapped TiO_2_ NPs. The condensed nuclei and apoptotic cells were indicated by arrows. In the control group, the cells exhibit normal morphology with intact nuclei. So, the staining results support the cytotoxic results obtained.

### 3.5. Antioxidant Markers

**Figure 5:**
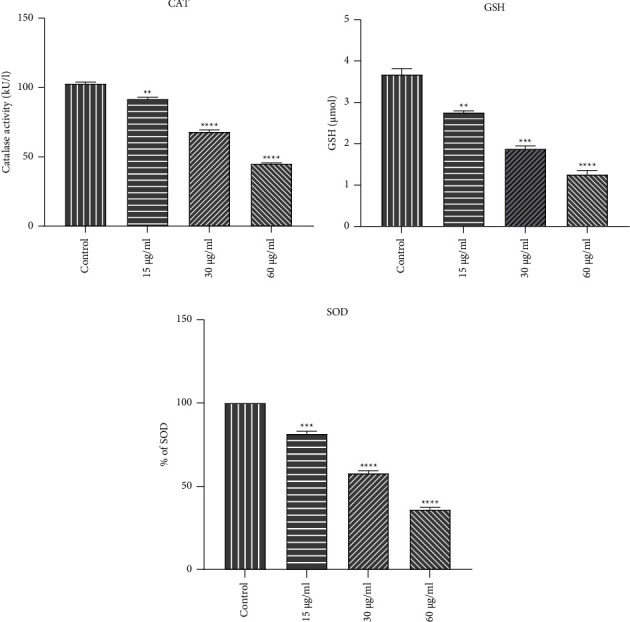
(a) CAT, (b) GSH, (c) SOD. Representation of the above activity between control and treated cells. When compared to control cells, the level of catalase was substantially lower in the treated cells. Results were subjected to one-way ANOVA (Tukey multiple comparison test) revealed that there was a significant difference between control and treated cells. In comparison with control cells, treated cells demonstrated high significance (^*∗∗∗∗*^*P* < 0.0001, ^*∗∗∗*^*P* ≤ 0.001 and ^*∗∗*^*P* ≤ 0.01).

To understand the homeostatic status of cells in the exposure of brucine entrapped TiO_2_ NPs, the investigation of oxidative markers is predominant one. For that purpose, the major factors such as catalase, reduced glutathione, and superoxide dismutase were determined.  Figure 5(a) showed the catalase assay. The graph showed a gradual decrease in catalase activity as the dose increased. The control group showed maximum catalase activity, whereas in treated groups 91.677 ± 1.39, 68.02 ± 1.39, and 45.09 ± 0.58 for 15, 30, and 60 *µ*g/mL, respectively. In reduced glutathione assay, the level of GSH was decreased constantly in a dose-dependent manner. Control showed 3.67 ± 0.14 and in treated group 2.75 ± 0.04, 1.87 ± 0.07, and 1.25 ± 0.09 was observed for 15, 30, and 60 *µ*g/mL, respectively (Figure 5(b)). Similarly, in the estimation of SOD, a significant decrease was observed in 15, 30, and 60 *µ*g/mL as 81.52 ± 1.52, 57.84 ± 1.47, and 35.91 ± 1.37, respectively (Figure 5(c)). The dose-dependent decrease in antioxidant levels such as CAT, GSH, and SOD denotes that brucine-entrapped TiO_2_ NPs manifested a strong cytotoxic effect in the HeLa cell line.

## 4. Discussion

The traditional method of cancer treatment possesses low specificity with severe side effects and multidrug resistance which increases the demand for alternative therapy. Drugs derived from natural sources can be used both as preventive and therapeutic, resulting in responsiveness for low dosage and autophagy-induced apoptosis for high dosage, but phytocompound delivery is a major challenge. Several studies have shown chemotherapeutic drugs and phytochemicals delivered by nanoparticle are effective both as therapeutic and chemo sensitizing agent.

Brucine, a weak-base indole alkaloid possesses anti-inflammatory, anti-snake venom, antioxidant, and analgesic activity [28–30]. Also exerted cytotoxic and antiproliferative MCF-7 [31], HepG2 [32], SMMC-7221 [33], and multiple myeloma RPMI 8226 [34]. Its therapeutic applicability in the treatment of cancer is constrained by its high toxicity, poor water solubility, brief half-life, and low toxic dose for intravenous usage. In order to prevent above challenges and improve drug interaction with target cells, the current investigation was carried out by coating brucine on titanium oxide nanoparticle. Several studies suggest that TiO_2_ NPs may significantly reduce tumour cell growth and boost the triggering of apoptosis. Because they quickly pass through cell membranes and exhibit excellent therapeutic efficacy. Based on this hypothesis, a titanium oxide nanoparticle was loaded with brucine and observed for its anticancer activity in the HeLa cell line [35, 36].

The synthesis of brucine-entrapped TiO_2_ NPs was validated by several characterization techniques. The absorption peak at 266 nm reveals the formation of TiO_2_ NPs. While the signal shifted from 260 nm to 302 nm eventually to 647 nm could be due to the coating of Brucine [37, 38]. In the FT-IR spectrum, the band at 3421 cm^−1^ correlates to alcoholic groups [39]. The C-H stretching vibrations of saturated carbon were denoted by peaks at 2841, 2867, and 2927 cm^−1.^ These stretching vibrations are in consistent with the FT-IR of brucine reported earlier [40]. The absorption band at 1653 cm^−1^ is assigned to carbonyl –C=O stretching vibrations of brucine. Altogether, this indisputably proves the effective conjugation of brucine to TiO NPs, and the results observed here are in agreement with earlier published data [41]. The C-N stretching vibrations of amine were denoted by the peaks at 1030 cm^−1^ [39]. The broad absorption band between 400 cm^−1^ to 900 cm^−1^ notably 648 cm^−1^ and 529 cm^−1^ attributed to Ti-O-Ti and Ti-O linkages and also reveals that Ti-O is at the anatase phase [37, 42–44]. The average size of brucine-entrapped TiO_2_ NPs was 100.0 nm with a polydispersity index of 0.320, as revealed in DLS. With an average size of 100 nm, TiO_2_ NPs can effectively offer a nanodrug delivery platform, hence improving the drug's appropriate solubility in the targeted cells. The results of the SEM analysis showed that certain portions of the nanoparticles were ellipsoidal in shape while the others had tetragonal shapes that were distributed unevenly in the range of 117.6, 123.2,147.5, and 154.6 nm [45]. The SEM results further confirm the anatase phase of TiO NPs since the anatase phase of TiO NPs occur in ellipsoidal form [46]. Furthermore, SEM results were also consistent with DLS. From EDAX data, the Ti signal was observed in the weight % of 38.94, atomic % of 16.22, and the O signal in the weight % of 42.57, atomic % of 53.08, ensuring the presence of TiO [44]. The C signal with weight % of 18.49, atomic % of 30.70 refers to carbon. Therefore, the EDAX results strongly correlate with the presence of carbonyl stretches of brucine in the FT-IR spectrum.

Brucine-loaded TiO_2_-treated cells caused a decrease in cell viability of the HeLa cell line in a dose-dependent manner with LC_50_ at 30 *µ*g of brucine loaded TiO NPs. Even at a low dosage 1.9 *µ*g/mL brucine-entrapped TiO_2_ NPs showed 17.48% inhibition. Phyto-modified titanium nanoparticles showed higher efficacy for anticancer activity due to the synthesis of superoxide radicals in cancer cells which was confirmed by research against A549 and KB cell line [47]. Our results also were consistent with DOX-TiO_2_ nanocomposites that increased lethality with increasing concentrations of DOX in a dose-dependent manner due to endocytosis, a common characteristic of nano-based drug delivery [48]. Morphological changes of shrinkage, rupturing, rounding, and detachment of cells were observed in treated cells which was further evaluated by DAPI staining to confirm apoptosis by blebbing and nuclear condensation. The external and internal pathways trigger the process of apoptosis in case of cancer cells. The inhibition of cervical cancer progression is accomplished by influencing the activity of many signalling pathways, leading to the regulation of genes associated with cell cycle and death [49–51].

During normal cellular activity, ROS is produced in less amounts. Apoptosis via oxidative stress can damage biological macromolecules such as proteins, lipids, and DNA [52]. Increased production of ROS within cells upon exposure to TiO_2_ NP coated with brucine could have been a contributing factor to the anticancer impact observed in cervical cancer cells. It also induced oxidative stress by raising ROS and lowering glutathione levels, which leads to the induction of apoptosis through the p53, survivin, Bax/Bcl-2, and caspase pathways which were similar to platinum-coated drugs. As a substrate for enzymatic antioxidants, glutathione is the endogenous antioxidant that shields cells from oxidative damage [53]. After being exposed to brucine-TiO nanoparticles for 24 hours in the current investigation, cancer cells displayed low glutathione levels. This impairs the cellular antioxidant defense mechanism, causing damage and ultimately leading to the death of these cells [49–51]. As a result, cancer cells were unable to properly remove ROS, hydrogen peroxide, and metabolites.

According to our findings, antioxidant enzyme activity, CAT, and SOD can be decreased by brucine-TiO Np. In relation to the significance of CAT activity in cancer, it has been shown that CAT activity inhibition significantly raises the level of oxidative stress and hydrogen peroxide, which kill cancer cells [54]. Similarly, SOD is also reduced with increased ROS and reduced glutathione which consistent with the results of other studies [55, 56]. Thus, it confirms the oxidative stress induced cell death of HeLa cells by brucine-TiO NPs.

## 5. Conclusion

The present study sheds insights on the anticancerous activity of brucineentrapped TiO_2_ NPs in the HeLa cell line. The peaks noticed in the UV spectrum and FT-IR established the formation of TiO_2_ NPs and entrapment of brucine to TiO_2_ NPs, respectively. The average size of brucine-entrapped TiO_2_ NPs was 100.0 nm in DLS analysis. SEM and EDAX showed ellipsoidal to tetragonal-shaped NPs with signatures of Ti, O, and C peaks. The MTT assay demonstrated an inhibition in cell proliferation, which is consistent with the trypan blue exclusion assay, phase contrast microscopic analysis, and fluorescence staining. In addition, decreased antioxidant markers in the treated cells may lead to apoptosis, which also proves the cytotoxic potential of brucine-entrapped TiO_2_ NPs. The current findings suggest that brucine-entrapped TiO_2_ NPs could provide effective nanodelivery system for delivering bioactive compounds which could be an alternative to currently available cancer treatment strategies. Further research is needed to explore the nanopharmacological capabilities and mechanism of action of brucine-entrapped TiO_2_ NPs.

## Data Availability

Data are made available upon request.
